# Supplementary figures and images for: Beyond the new normal: Assessing the feasibility of vaccine-based suppression of SARS-CoV-2
Source: PLoS One. 2021 Jul 16;16(7):e0254734. doi: 10.1371/journal.pone.0254734 (PMC8284637; doi:10.1371/journal.pone.0254734)

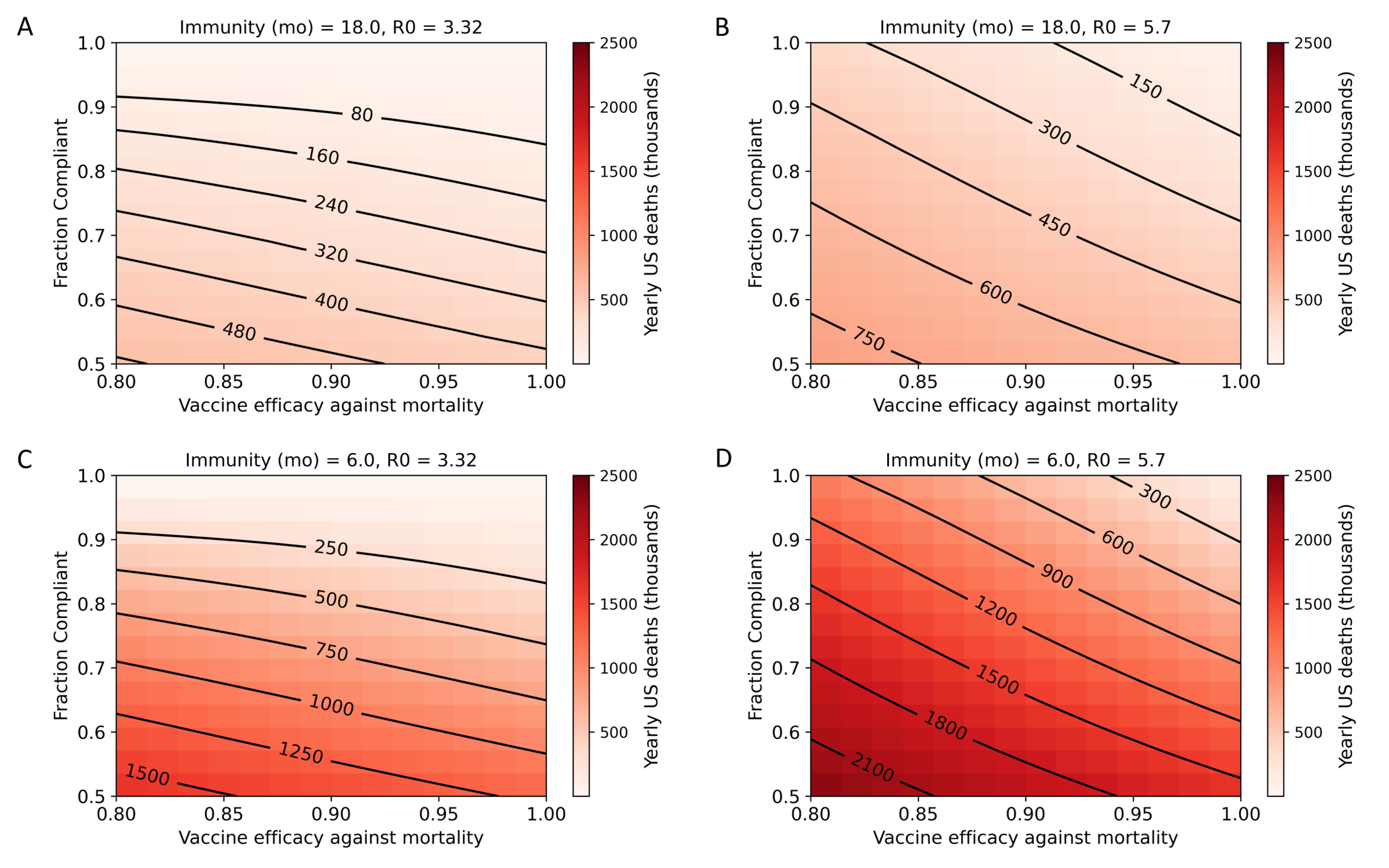

Supplement: S1 Fig — This figure is parallel to Fig 1 in the main text but explores four sets of parameters for the duration of natural immunity and R0. Panels represent four possible scenarios: A) R0 of 3.32 with an 18-month duration of natural immunity, B) R0 of 5.7 with an 18-month duration of immunity, C) R0 of 3.32 with a 6-month duration of immunity, D) R0 of 5.7 with a 6-month duration of immunity. (TIF) [file pone.0254734.s001.tif]

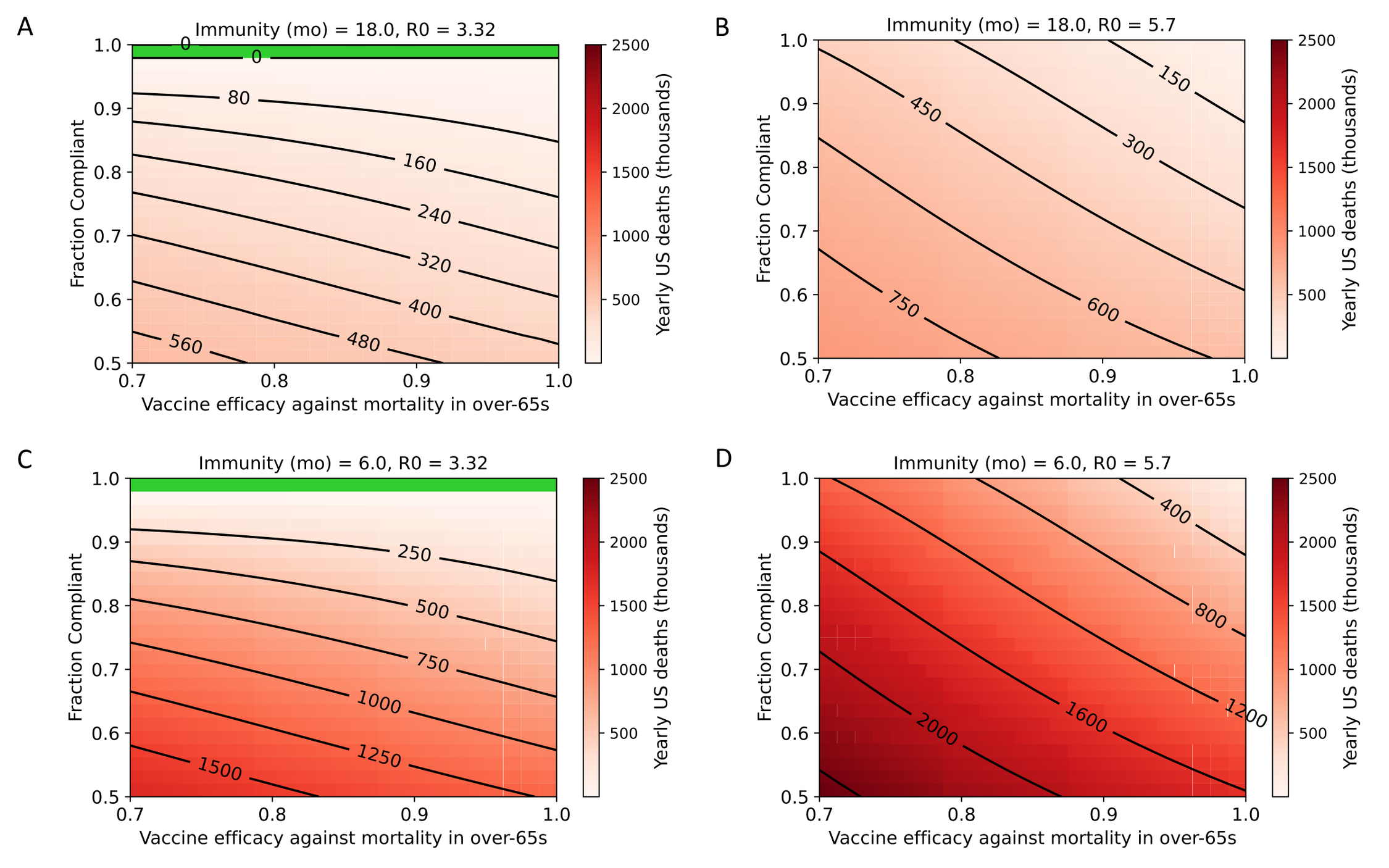

Supplement: S2 Fig — This figure is parallel to Fig 2 in the main text but explores four sets of parameters for the duration of natural immunity and R0. (TIF) [file pone.0254734.s002.tif]

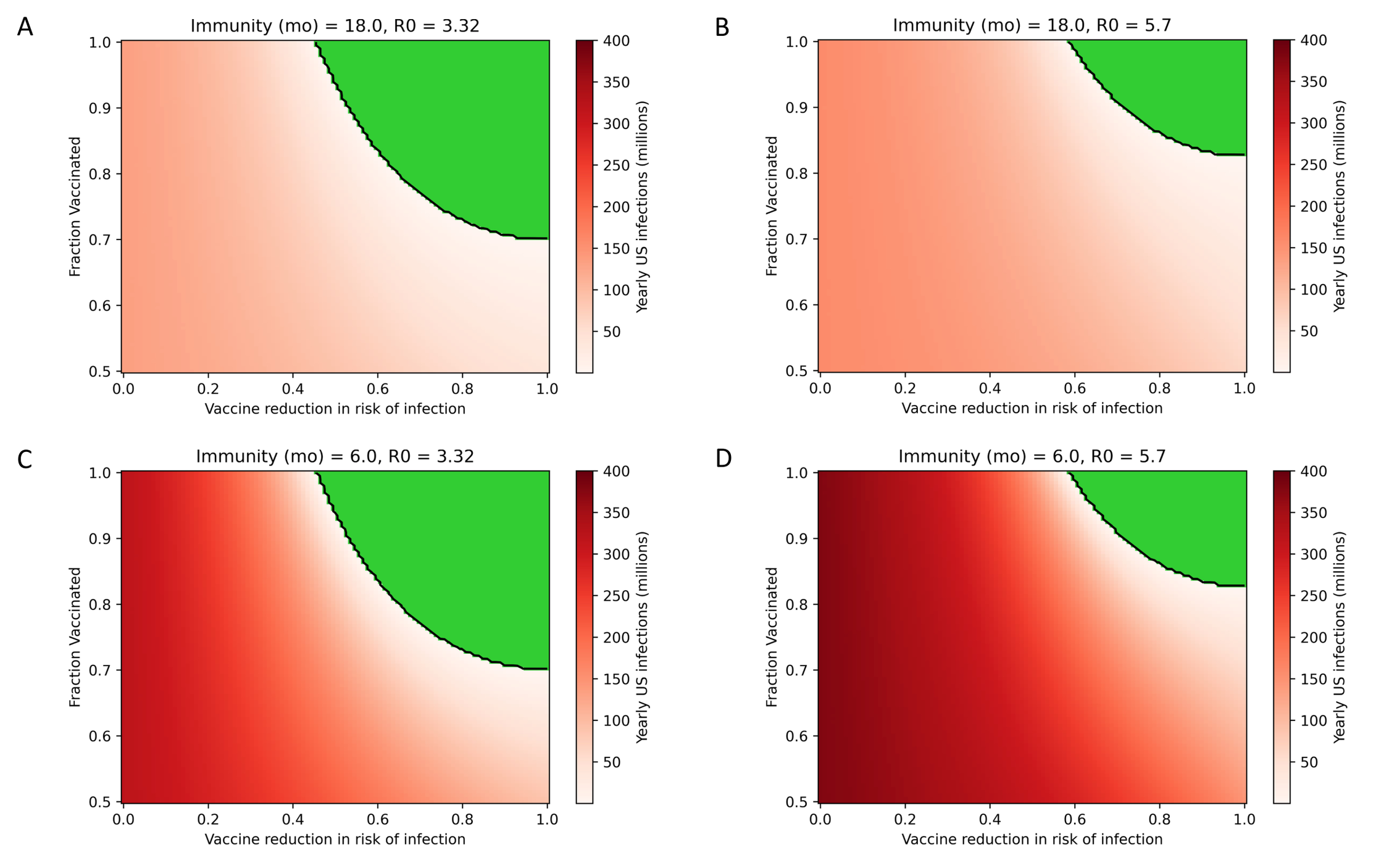

Supplement: S3 Fig — This figure is parallel to Fig 3 in the main text but explores four sets of parameters for the duration of natural immunity and R0. Green region represents regime in which SARS-CoV-2 is eliminated in the population and yearly infections approach zero. (TIF) [file pone.0254734.s003.tif]

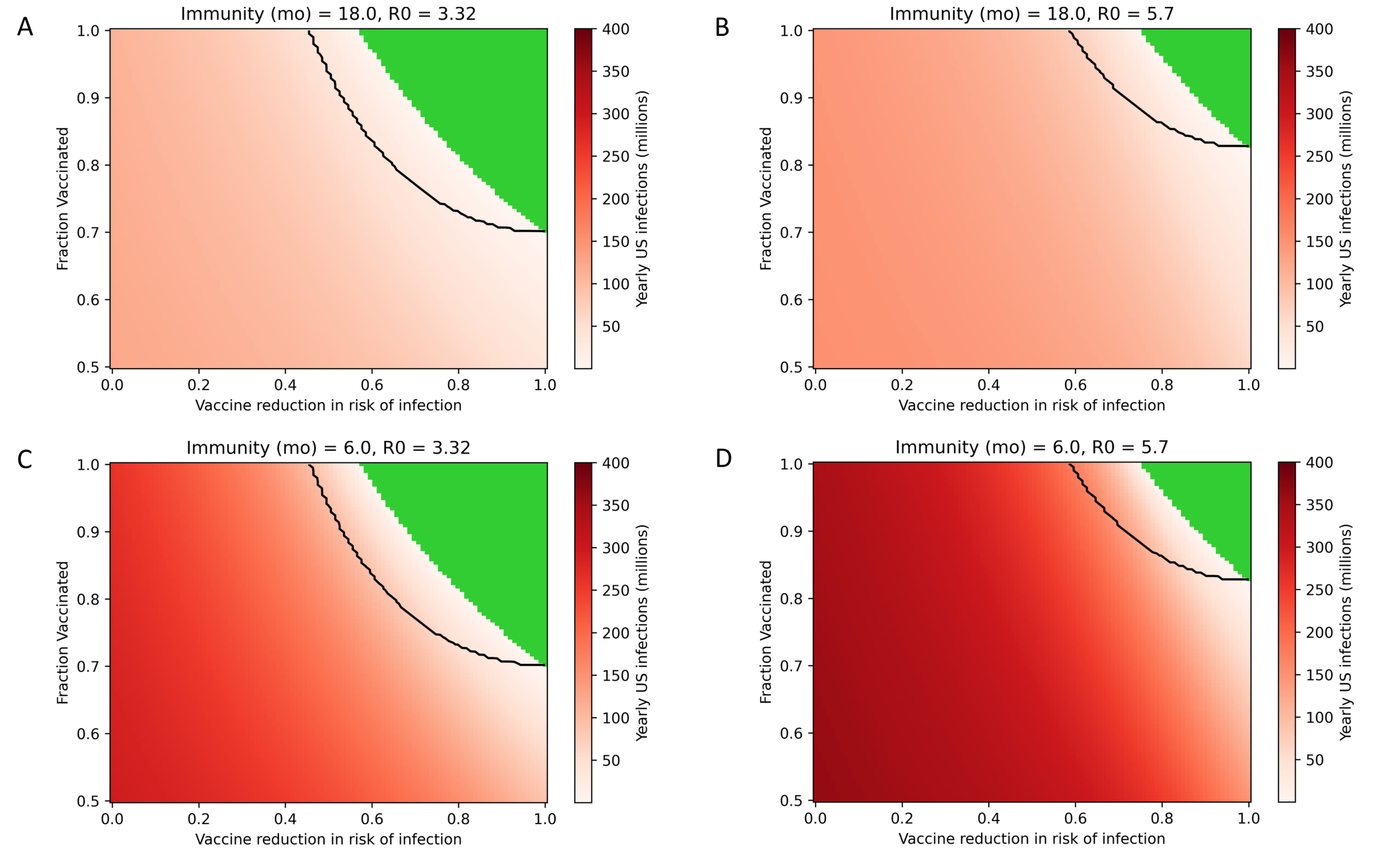

Supplement: S4 Fig — This figure is parallel to Fig 4 in the main text but explores four sets of parameters for the duration of natural immunity and R0. Black lines outline the suppression space for a vaccine that prevents infection and transmission to an equal degree, as shown in S3 Fig. Green region represents cases where suppression under this vaccine is achieved, with virtually zero yearly infections. (TIF) [file pone.0254734.s004.tif]

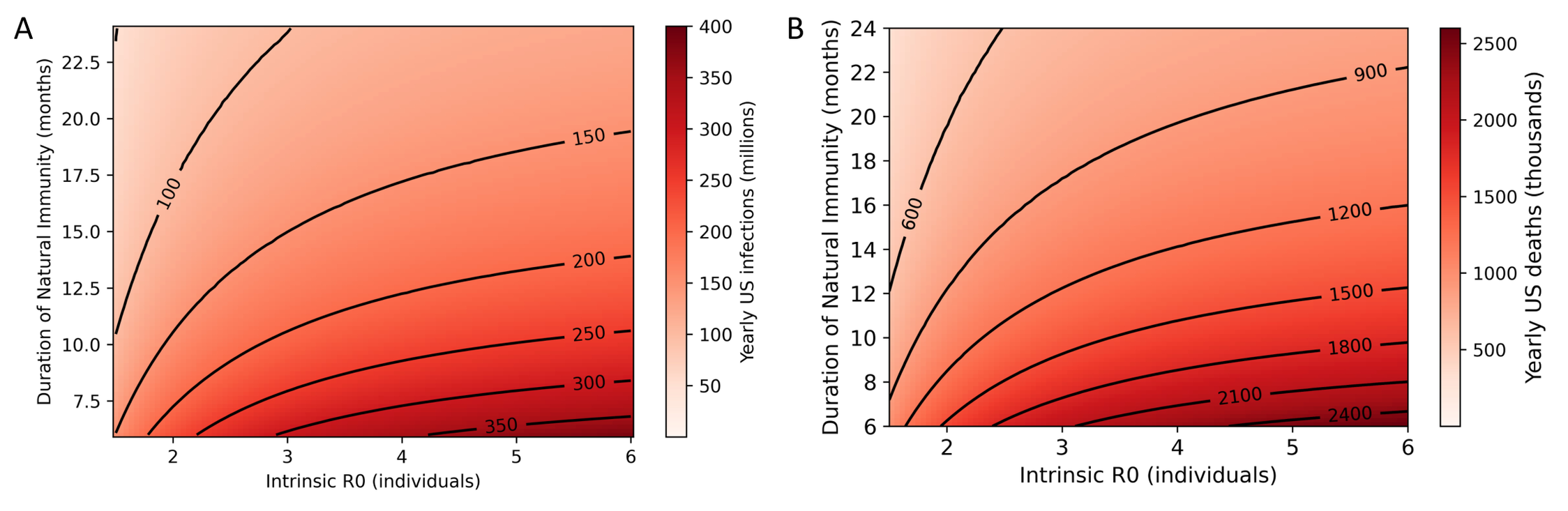

Supplement: S5 Fig — US yearly infections (A) and COVID-19 deaths (B) are predicted at steady-state for a variety of R0 and duration of natural immunity estimates. Extensive disease and mortality burdens are expected under all endemic scenarios. (TIF) [file pone.0254734.s005.tif]

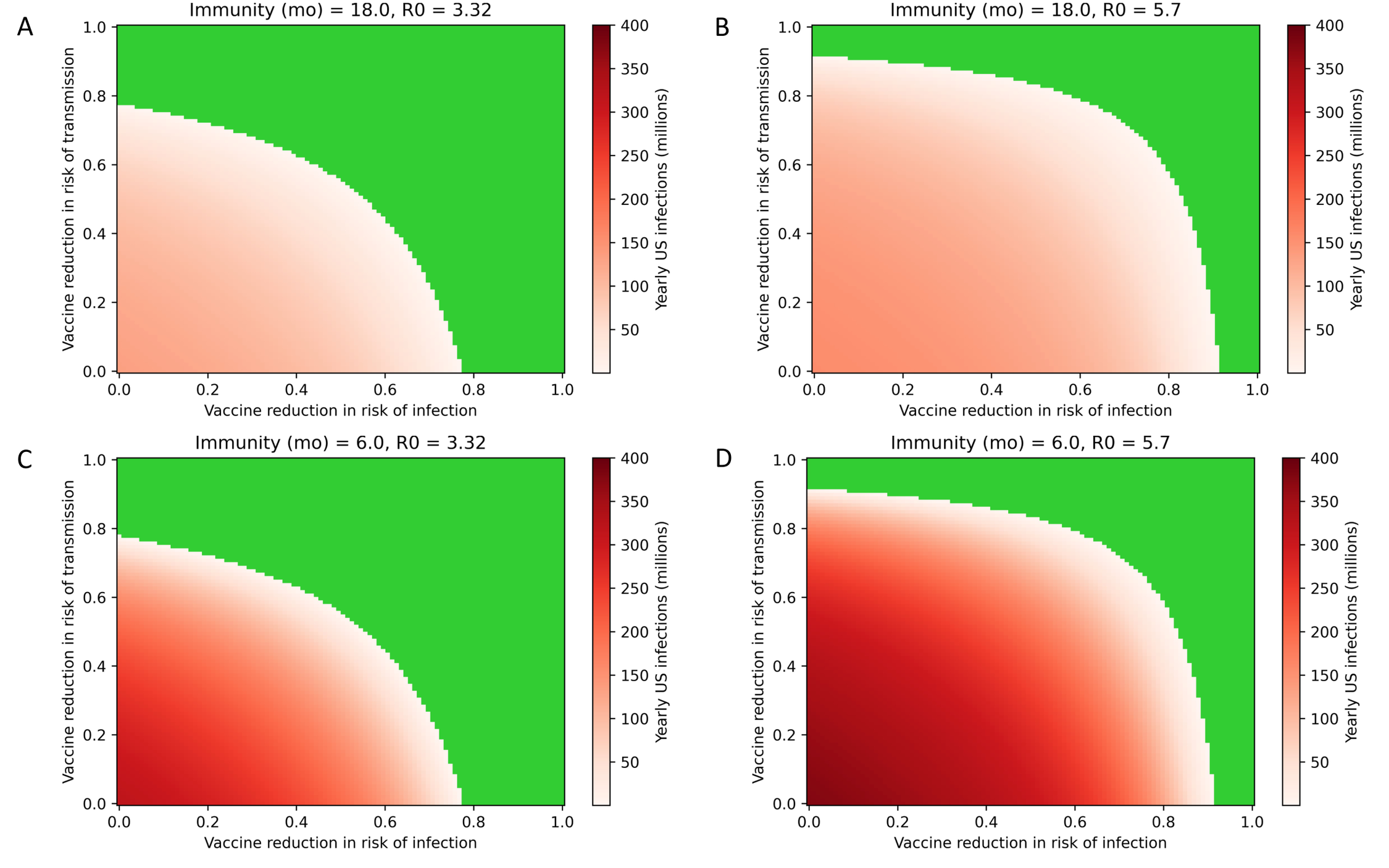

Supplement: S6 Fig — Vaccine efficacy against transmission is equally impactful compared to vaccine efficacy against infection and determines success or failure to achieve eradication in many cases. (TIF) [file pone.0254734.s006.tif]

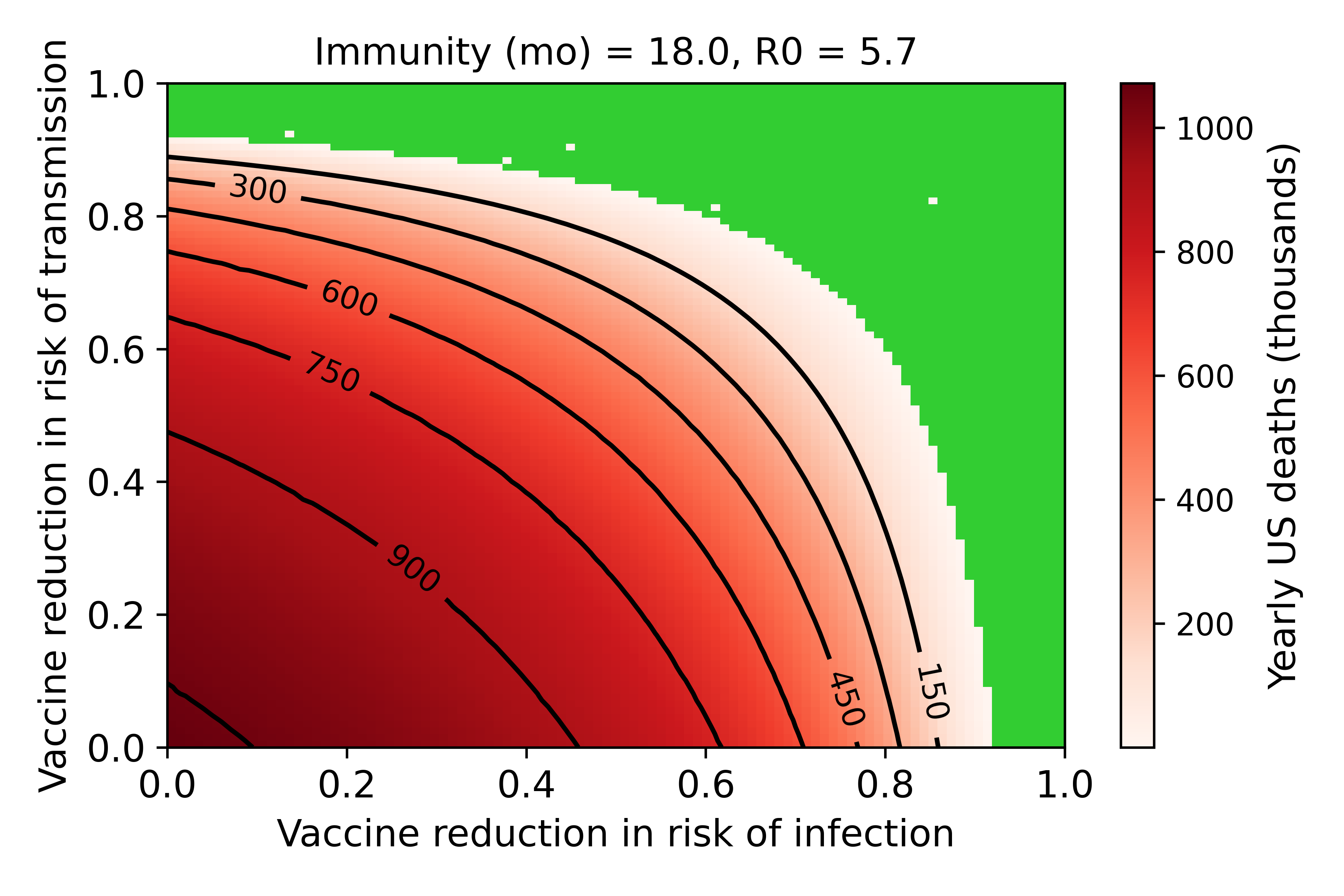

Supplement: S7 Fig — In this figure, 90% of Americans are assumed to be vaccinated and the vaccine is assumed to have an age-independent 95% efficacy against mortality. (TIF) [file pone.0254734.s007.tif]

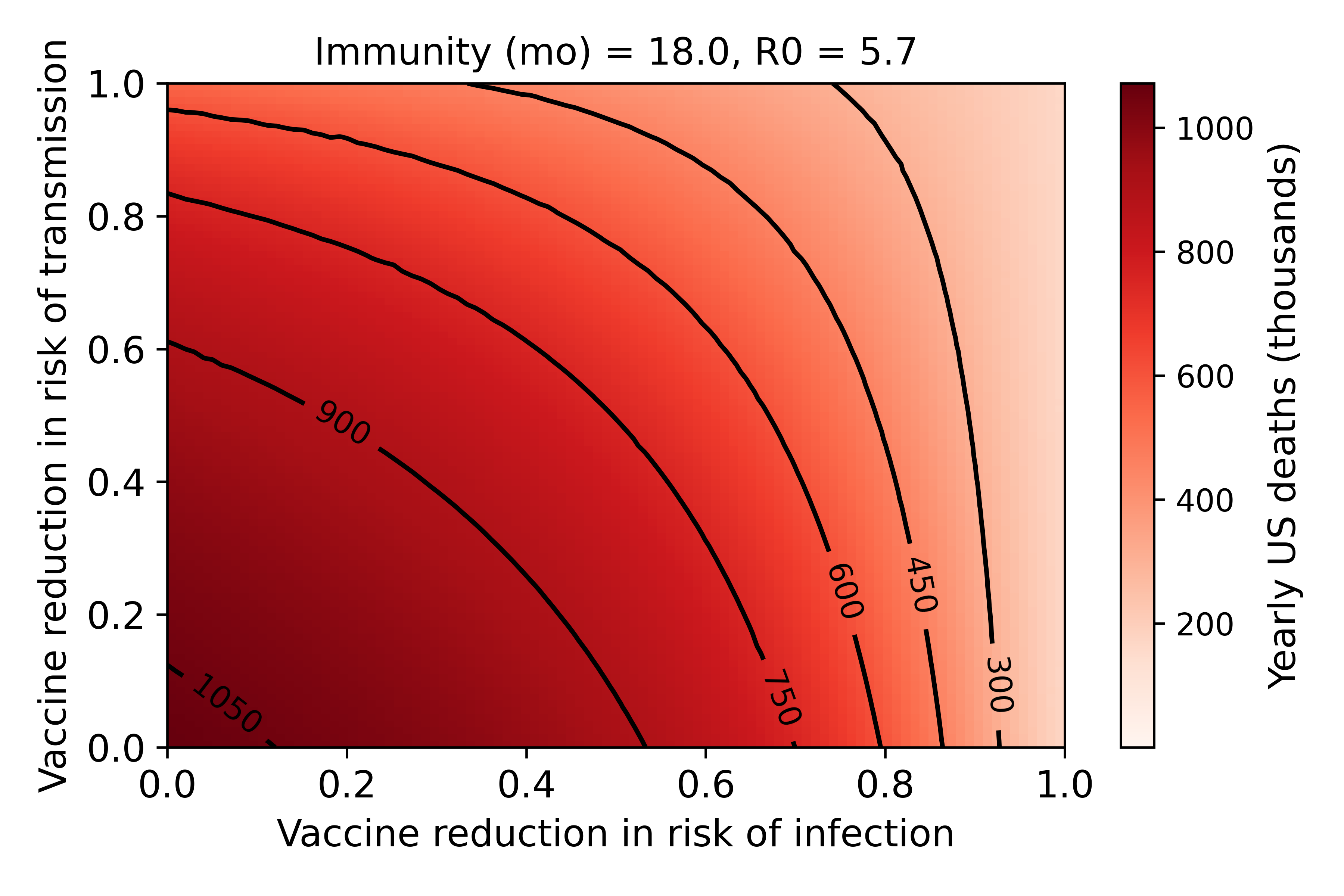

Supplement: S8 Fig — In this figure, 70% of Americans are assumed to be vaccinated and the vaccine is assumed to have an age-independent 95% efficacy against mortality. At this R0, disease suppression is impossible with only 70% vaccine compliance. (TIFF) [file pone.0254734.s008.tiff]

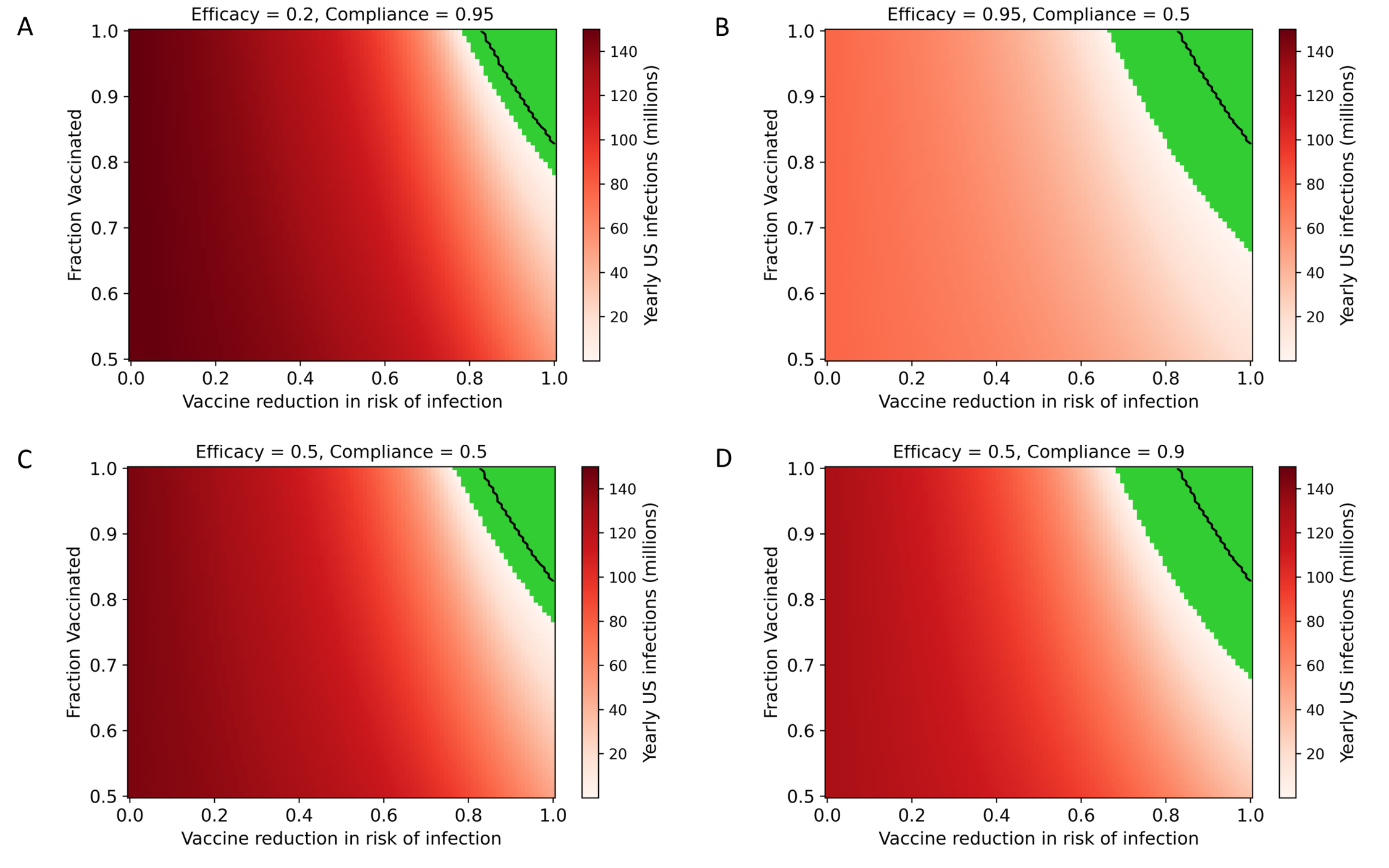

Supplement: S9 Fig — Black lines outline the eradication space for the vaccine alone. This figure is parallel to Fig 4; the vaccine is assumed to have no effect on transmission, the R0 is assumed to be 5.7, and the duration of natural immunity is 18 months. Black lines outline the eradication space for the vaccine alone. In the figure panels, four example complementary interventions are explored: A) a compatible, competing vaccine achieving 50% reduction in risk of infection and 50% compliance in the population; B) universal masking, which reduces the risk of infection by 50% and reaches 90% compliance; C) a passive intervention, such as improving indoor ventilation, which impacts 95% of the population but has a small 20% impact on risk of infection; and D) a highly effective (95%) intranasal prophylactic that 50% of the population uses. (TIF) [file pone.0254734.s009.tif]

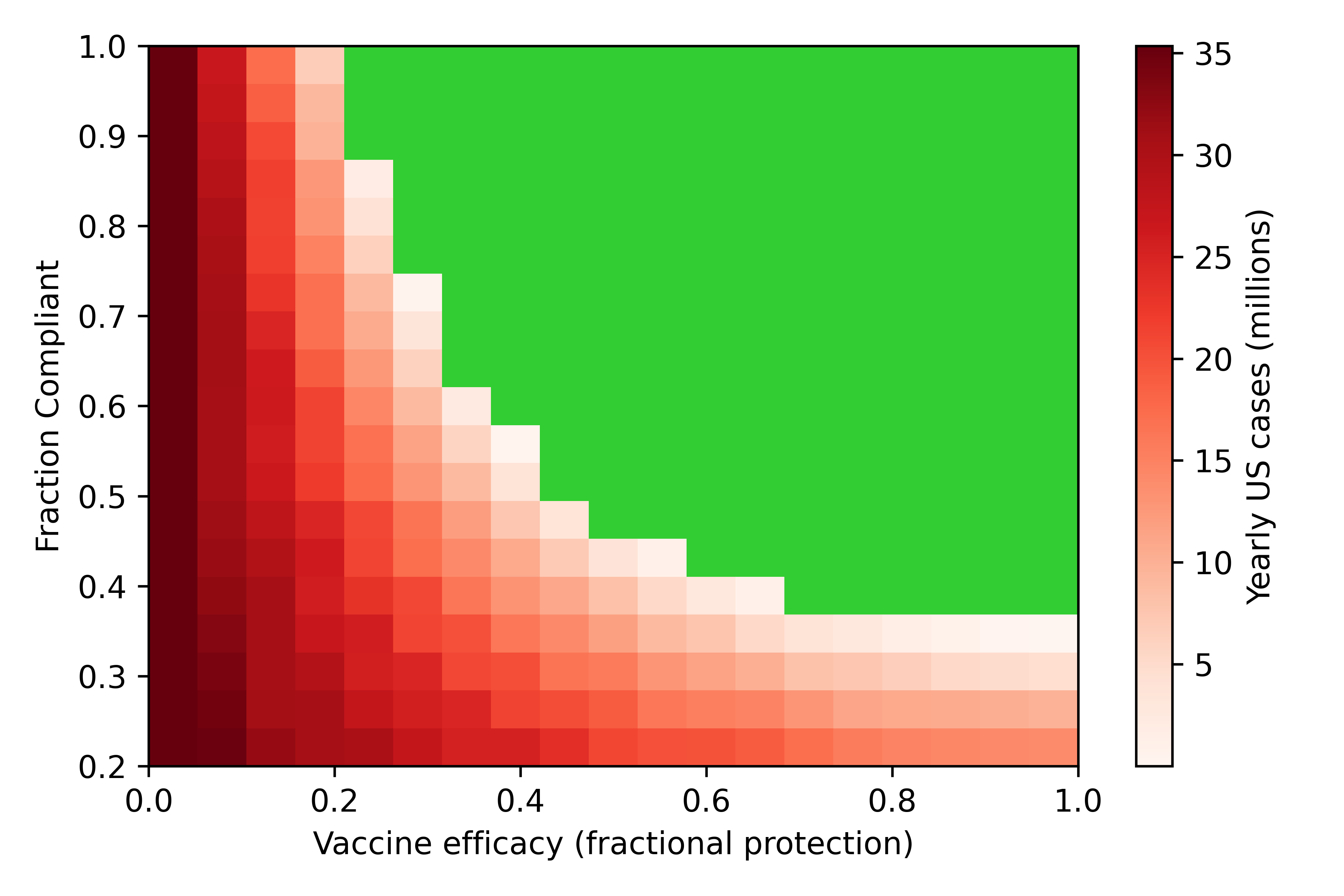

Supplement: S10 Fig — Green region represents successful vaccine-based eradication based on an SEIR model for influenza [72]. (TIF) [file pone.0254734.s010.tif]
