# Supplementary material for: Beyond the new normal: Assessing the feasibility of vaccine-based suppression of SARS-CoV-2
Source: PLoS One. 2021 Jul 16;16(7):e0254734. doi: 10.1371/journal.pone.0254734 (PMC8284637; doi:10.1371/journal.pone.0254734)
Supplement: S1 File — (DOCX) [file pone.0254734.s011.docx]

**Supplemental Methods and Results**

*Estimation of AstraZeneca vaccine impact on transmission risk in vaccinated infected individuals*

Based on the data summarized in Table S1, we estimated that the AstraZeneca vaccine reduced the risk of symptoms given SARS-CoV-2 infection by 22%. Asymptomatic SARS-CoV-2 carriers have been demonstrated to transmit at a 42% reduced frequency relative to symptomatic carriers [1]. A 42% reduction in risk of transmission given infection in the 22% of vaccinated infected individuals who are subsequently asymptomatic translates to a 9% overall reduction in risk of transmission upon infection. This estimated reduction in transmission upon infection combined with the 49.5% reduction in risk of infection (as reported by AstraZeneca for the standard dose / standard dose regimen [2]) translates to an aggregated 54% reduction in risk of transmission in vaccinated individuals.

**S1 Table:** Distribution of asymptomatic and symptomatic SARS-CoV-2 carriers in the standard dose regimen of the AstraZeneca vaccine [2]

|  | Vaccinated | Placebo |
| --- | --- | --- |
| Asymptomatic | 41 | 42 |
| Symptomatic | 74 | 197 |

*Herd immunity strategy results in extensive endemic disease*

Fringe groups have proposed allowing COVID-19 to spread freely among the general population with the hope that natural immunity will eventually slow the spread of disease. Although the SEIRS model demonstrates that a strategy entirely dependent on natural immunity would eventually lead to stabilization of SARS-CoV-2 transmission, the steady-state that is reached involves catastrophic disease burden and mortality (Figure S5). Although estimates of the R_0_ and duration of immunity of SARS-CoV-2 vary, optimistic estimates for these parameters still result in more than 100,000 COVID-19 deaths annually. Under no relevant conditions does the pandemic extinguish itself without intervention. Our best estimate for R_0_ is 5.7 based on CDC data and for the duration of immunity is 18 months based on the time-to-baseline for anti-SARS-CoV-2 antibodies post-infection. Under these conditions, the model predicts 176 million annual US infections and 1.2 million annual US deaths if no action is taken to contain SARS-CoV-2.

**References**

1. Nogrady B. What the data say about asymptomatic COVID infections. Nature. 2020;587: 534–535. doi:10.1038/d41586-020-03141-3

2. Voysey M, Costa Clemens SA, Madhi SA, Weckx LY, Folegatti PM, Aley PK, et al. Single Dose Administration, And The Influence Of The Timing Of The Booster Dose On Immunogenicity and Efficacy Of ChAdOx1 nCoV-19 (AZD1222) Vaccine. Rochester, NY: Social Science Research Network; 2021 Feb. Report No.: ID 3777268. Available: https://papers.ssrn.com/abstract=3777268
